# Seven Immune-Related Genes' Prognostic Value and Correlation with Treatment Outcome in Head and Neck Squamous Cell Carcinoma

**DOI:** 10.1155/2023/8533476

**Published:** 2023-04-20

**Authors:** Rui Mu, Yuehong Shen, Chuanbin Guo, Xinyun Zhang, Hongyu Yang, Huijun Yang

**Affiliations:** ^1^Stomatology Center, The Institute of Stomatology, Peking University Shenzhen Hospital, Shenzhen Peking University-The Hong Kong University of Science and Technology Medical Center, Shenzhen, China; ^2^Guangdong Provincial High-Level Clinical Key Specialty, Shenzhen, China; ^3^Guangdong Province Engineering Research Center of Oral Disease Diagnosis and Treatment, Shenzhen, China; ^4^Department of Oral and Maxillofacial Surgery, Peking University School and Hospital of Stomatology, Beijing, China; ^5^School of Clinical Medicine, The Zhuhai Campus of the Zunyi Medical University, Zhuhai, China

## Abstract

**Background:**

Head and neck squamous cell carcinoma (HNSCC) is a growing concern worldwide, due to its poor prognosis, low responsiveness to treatment, and drug resistance. Since immunotherapy effectively improves HNSCC patients' survival status, it is important to continuously explore new immune-related predictive factors to accurately predict the immune landscape and clinical outcomes of individuals suffering from HNSCC.

**Methods:**

The HNSCC transcriptome profiling of RNA-sequencing data was retrieved from TCGA database, and the microarray of GSE27020 was obtained from the GEO database for validation. The differentially expressed genes (DEGs) between HNSCC and normal samples were identified by multiple test corrections in TCGA database. The univariate and multivariate Cox analyses were performed to identify proper immune-related genes (IRGs) to construct a risk model. The Cox regression coefficient was employed for calculation of the risk score (RS) of IRG signature. The median value of RS was utilized as a basis to classify individuals with HNSCC into high- and low-risk groups. The Kaplan-Meier (K-M) survival analysis and receiver operating characteristic (ROC) curves were employed for the identification of the prognostic significance and precision of the IRG signature. The signature was also evaluated based on clinical variables, predictive nomogram, mutation analysis, infiltrating immune cells, immune-related pathways, and chemotherapeutic efficacy. The protein-protein interaction (PPI) network and functional enrichment pathway investigations were utilized to explore possible potential molecular mechanisms. Finally, the hub gene's differential mRNA expression levels were evaluated by means of the Gene Expression Profiling Interactive Analysis (GEPIA), and the Human Protein Atlas (HPA) was utilized for the validation of their translational levels.

**Results:**

Collectively, 1593 DEGs between HNSCC and normal samples were identified, of which 136 IRGs were differentially expressed. Then, the 136 immune-related DEGs were mostly enriched in the cytokine-related signaling pathways by GO and KEGG analyses. After that, a valuable signature based on seven genes (*DKK1*, *GAST*, *IGHM*, *IL12RB2*, *SLURP1*, *STC2*, and *TNFRSF4*) was designed. The HNSCC patients into the low-risk group and the high-risk group were divided by using the median RS; the HNSCC patients in the high-risk group had a worse survival than those in the low-risk group. The risk signature was verified to be an independent predictive marker for HNSCC patients. Meanwhile, the RS had the largest contribution to survival of these patients based on the predictive nomogram. In addition, the low-risk HNSCC patients exhibited significantly enriched immune cells, along with an association with high chemosensitivity.

**Conclusion:**

The constructed gene signature can independently function as a predictive indicator for the clinical features of HNSCC patients. The low-risk HNSCC subjects might benefit from immunotherapy and chemotherapy.

## 1. Introduction

As the sixth most prevalent type of malignancy, head and neck squamous cell carcinoma (HNSCC) is the seventh main cause of cancer-related mortalities globally [1]. A study conducted in the United States predicted that by 2022, approximately 66,470 new HNSCC cases would arise and 15,050 HNSCC-related deaths would occur [2]. Despite steady advancements in relevant medical treatments, like surgery, radiotherapy, and chemotherapy, the five-year survival of individuals with HNSCC has not significantly improved [3]. Therefore, finding new and innovative novel prognostic factors for HNSCC patients is an urgent need.

HNSCC is considered an immunodeficiency disease. The main mechanisms underlying the disease include the induction of immune tolerance, local immune escape, and the destruction of T-cell signals [4]. The immune microenvironment of HNSCC has been widely studied [5, 6]. For instance, the human leukocyte antigen (HLA) is responsible for the vital function of transmitting signals between tumor antigen peptides and killer T cells [7]. A previous study demonstrated that more than 50% of HNSCC patients had low HLA expression, with extensive lymph node metastasis and poor prognosis [8]. HNSCC tumor cells could also release chemical factors, to induce many immunosuppressive hematological cells to enter the immune microenvironment, thus suppressing the immune response [9].

Meanwhile, studying how HNSCC survival is linked to infiltrating immune cell proliferation and function could improve the survival of HNSCC patients [10–12]. Immunotherapy's effect on the clinical outcomes of individuals with HNSCC has also been intensively studied [13–15]. This is why the exploration of immune-related biomarkers to anticipate the clinical features of individuals with HNSCC is imperative. Recent studies demonstrated that immune-related biomarkers could affect the biological behavior of HNSCC as well as the status of patients. For instance, Yao et al.'s model consisted of four immune-related genes (IRGs), including *PVR*, *TNFRSF12A*, *IL21R*, and *SOCS1* [16]; Chen et al. constructed predictive model based on three IRGs (*SFRP4*, *CPXM1*, and *COL5A1*) [17]; and Zhang et al. established a model based on six IRGs (*PLAU*, *STC2*, *TNFRSF4*, *PDGFA*, *DKK1*, and *CHGB*) for the prognostic prediction of HNSCC [18]. Although these studies have constructed a proper model to predict the prognosis of patients with HNSCC, the progression of HNSCC is complexity and uncertainty. Therefore, to date, reliable and predictive biomarkers for identifying HNSCC are still limited; it is essential to continuously look for newly representative biomarkers.

During this research, a risk signature of seven immune-related genes was developed for accurately predicting the clinical outcomes of HNSCC subjects, which may provide an effective treatment strategy for these patients.

## 2. Methods

### 2.1. Dataset

The transcriptome profiling of RNA-sequencing (FPKM) was attained from The Cancer Genome Atlas (TCGA) (https://cancergenome.nih.gov/) containing 502 cancerous and 44 healthy tissues samples, along with the relevant clinical information for 528 HNSCC patients. In total, 2483 IRGs were procured from The ImmPort database (https://immport.niaid.nih.gov/) [19, 20]. Besides, the microarray and clinical information of GSE27020 containing 109 HNSCC samples were provided by the Gene Expression Omnibus (GEO) (https://www.ncbi.nlm.nih.gov/gds/) for authentication.

### 2.2. Development of the IRG-Based Signature

The differentially expressed genes (DEGs) between cancerous and healthy samples were identified with the help of TCGA database after multiple test corrections by false discovery rate (FDR) < 0.05 and |log fold change (FC)| > 2 [21]. Then, screening the intersections of these DEGs with IRGs was carried out. Univariate Cox proportional hazards regression analysis was employed for the determination of the predictive ability of IRGs for the overall survival (OS) of individuals with HNSCC with the aid of the “survival” package in R [22]. The genes with a threshold of *P* < 0.05 were subjected to further evaluation using multivariate Cox regression analysis [23]. Then, the expression levels of the hub genes were compared for further exploring their expression features in normal and HNSCC tumor tissues. Subsequently, the calculation of the IRG-based signature-related risk score (RS) was done using the Cox regression coefficient and gene expression formula given below:

(1) $$\begin{array}{c} \text{RS}=\overset{N}{\underset{i=1}{\sum }}\left(\text{Expi}*\text{Co}‐\text{eff}\right) \end{array}$$

*N*, Expi, and Co-eff indicate signature gene number, gene expression levels, and regression coefficient values, respectively. Using the median value of RS as a criterion, the individuals with HNSCC were classified into the low- and high-risk groups.

### 2.3. Prognosis Prediction by the IRG-Based Signature

For the verification of the IRG-based signature's prognostic performance, the signature's impact on OS in both risk groups was subjected to comparison by Kaplan-Meier (K-M) survival analysis utilizing the “survival” package in R software, followed by the area under the curve (AUC) of the receiver operating characteristic (ROC) curves to assess IRG-based signature's accuracy through the “survival ROC” package in R software [24]. Subsequently, the GEO database (GSE27020) was used for external validation. It was also subjected to K-M survival analysis and ROC curves to identify the signature's prognostic value and precision. The RS distribution and survival status of individuals with HNSCC in TCGA were constructed to further understand the prognostic capability of the signature.

### 2.4. Correlation Analysis

The association of IRG-based signature with clinical variables (age, pathological grade, gender, and tumor and TNM stages) was analyzed. Moreover, the clinical factors and robustness of the signature in predicting the OS were demonstrated by employing univariate and multivariate Cox regression analyses.

### 2.5. Construction of a Predictive Nomogram

Based on clinical variables, a nomogram was used to establishment a prognostic scoring system for predicting survival in HNSCC patients both in TCGA and GSE27020 databases.

### 2.6. Somatic Mutation Analysis

We obtained the somatic mutation profiles of all tumor samples from TCGA database and explored the mutation analysis for 528 patients. The R software “maftools” package was utilized to analyzed and visualized for mutation data of the low-risk group and the high-risk group.

### 2.7. Immune Microenvironment Analysis

Considering the involvement of infiltrating immune cells in the tumor microenvironment, the single-sample gene set enrichment analysis (ssGSEA) algorithm was utilized to evaluate the immune score of each HNSCC sample from TCGA and GSE27020 databases [25]. The different proportions of the infiltrating immune cells between the low- and high-risk groups were assessed by the Wilcoxon test. Moreover, the association between the RS and immune-related biological functions was performed for further exploring the underlining mechanisms. The gene expression profiles corresponding to samples of TCGA and GSE27020 databases were selected to perform the gene set variation analysis (GSVA).

### 2.8. Prediction of Clinical Application

The calculation of the half inhibitory concentration (IC50) of common chemotherapeutic agents was done, and the differences in the IC50 across the two risk groups were also evaluated for predicting the clinical application of the IRG-based signature both in TCGA and GSE27020 databases.

### 2.9. Molecular Mechanism Analysis

The STRING biological database (https://string-db.org/) was applied for extraction of the protein-protein interaction (PPI) network [26] as a mathematical representation of the physical contacts among differentially expressed IRGs linked to HNSCC patient survival. Thereafter, Gene Ontology (GO) and Kyoto Encyclopedia of Genes and Genomes (KEGG) pathway enrichment analyses were employed in determining the potential function of the immune-related DEGs [27].

### 2.10. Investigating the Expression of Hub Genes

The Gene Expression Profiling Interactive Analysis (GEPIA) (http://gepia.cancer-pku.cn/) was utilized for the investigation of the differential mRNA expression profiles of the hub genes in the IRG-based signature. Moreover, the Human Protein Atlas (HPA) (https://www.proteinatlas.org/) was employed for the purpose of validating the translational levels of these hub genes.

### 2.11. Statistical Analysis

R version 3.6.2 was utilized to conduct statistical analysis procedures. DEGs were compared with multiple test corrections with FDR < 0.05 and |logFC| > 2 were viewed as being dramatically dysregulated. The survival curves were estimated by using the K-M survival analysis and log-rank test between different groups. Clinicopathological features were compared by univariate and multivariate Cox regression analyses. The ssGSEA algorithm and Wilcoxon test were used to compare different proportions of the infiltrating immune cells between different groups. The *t*-test or Wilcoxon test for comparisons of two variables, and a *P* < 0.05 (two-side) was taken as a statistically significant value.

## 3. Results

### 3.1. Differential Gene Expression Analysis

**Figure 1:**
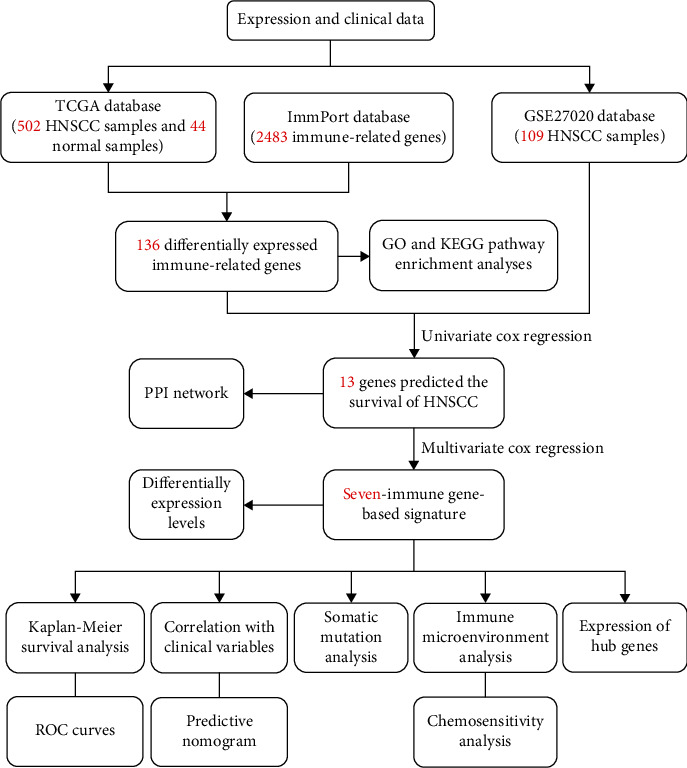
The workflow of the present study. TCGA: The Cancer Genome Atlas; HNSCC: head and neck squamous cell carcinoma; PPI: protein-protein interaction; GO: Gene Ontology; KEGG: Kyoto Encyclopedia of Genes and Genomes; ROC: receiver operating characteristic.

**Figure 2:**
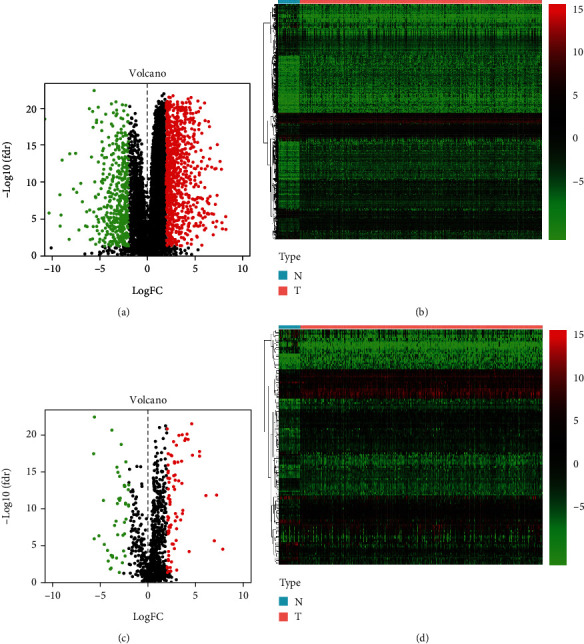
Analysis of differentially expressed genes. (a) Volcano plot of differentially expressed genes in HNSCC. Red dots represent upregulated genes, and green dots represent downregulated genes with statistical significance (FDR < 0.05, |logFC| > 2), while black dots represent the genes without differential significance. (b) Heatmap of differentially expressed genes in HNSCC tumor tissues. The colors from green to red represent differentially expressed genes with low to high expression levels. (c) Volcano plot of differentially expressed immune-related genes in HNSCC tumor tissues. (d) Heatmap of differentially expressed immune-related genes in HNSCC tumor tissues.

**Figure 3:**
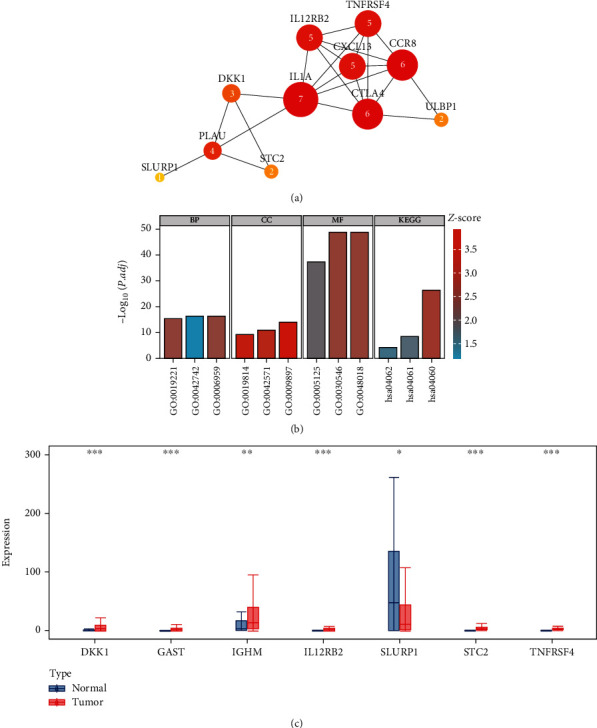
Immune-related differentially expressed genes analyzes. (a) PPI network of immune-related differentially expressed genes as predictors of prognosis of HNSCC patients. The Arabic numerals represent the degree of genes. (b) The GO and KEGG pathway analyses based on immune-related differentially expressed genes. (c) Comparison of the expression levels of the seven IRGs between normal tissues and tumor tissues. ^∗^*P* < 0.05,  ^∗∗^*P* < 0.01, and^∗∗∗^*P* < 0.001.

TCGA database was employed to retrieve the HNSCC RNA-sequencing data comprising 502 tumor samples and 44 healthy samples. Among these patients, 528 HNSCC subjects with gene expression profiles and clinical follow-up data were included. The workflow of this research is illustrated in Figure 1. In the differential gene expression analysis, 1593 DEGs between HNSCC and healthy samples were identified (Figures 2(a) and 2(b)), of which 136 IRGs were differentially expressed (Figures 2(c) and 2(d)). Among these genes, 13 genes were identified to predict survival in the univariate Cox regression analysis. To study their interactions, the STRING biological database was utilized to construct the PPI network, containing 11 nodes and 22 edges. Based on the degree of genes, *IL1A*, *CTLA4*, *CCR8*, *IL12RB2*, *TNFRSF4*, *CXCL13*, and *PLAU* appeared to be the core genes among these IRGs (Figure 3(a)). As for functional analysis in Figure 3(b), the 136 immune-related DEGs were mostly enriched in immune response/cytokine mediation (BP), immunoglobulin complex/external side of plasma membrane (CC), and cytokine activity/signaling receptor activator activity/receptor ligand activity (MF) by GO analysis. By KEGG pathway analysis, the genes were mostly enriched in cytokine-cytokine receptor interaction, viral protein interaction with cytokine and cytokine receptor, and chemokine signaling pathway.

### 3.2. Development and Verification of the IRG-Based Prognostic Signature

The detailed characteristics along with population demographics are given in Table 1. To develop a predictive IRG-based signature, seven IRGs were chosen after univariate and multivariate Cox analyses (Table 2). Meanwhile, the expression levels of the seven IRGs were further investigated. Compared with normal tissues, only *SLURP1* was downregulated in tumor tissues, while the expression levels of *DKK1*, *GAST*, *IGHM*, *IL12RB2*, *STC2*, and *TNFRSF4* were upregulated in tumor tissues (Figure 3(c)). Then, the calculation of the RS of this IRG-based signature was done as follows: RS = (0.006062^∗^*DKK*1) + (0.010886^∗^*GAST*) + (−0.000928^∗^*IGHM*) + (−0.051088^∗^*IL*12*RB*2) + (−0.001863^∗^*SLURP*1) + (0.025190^∗^*STC*2) + (−0.089341^∗^*TNFRSF*4).

**Figure 4:**
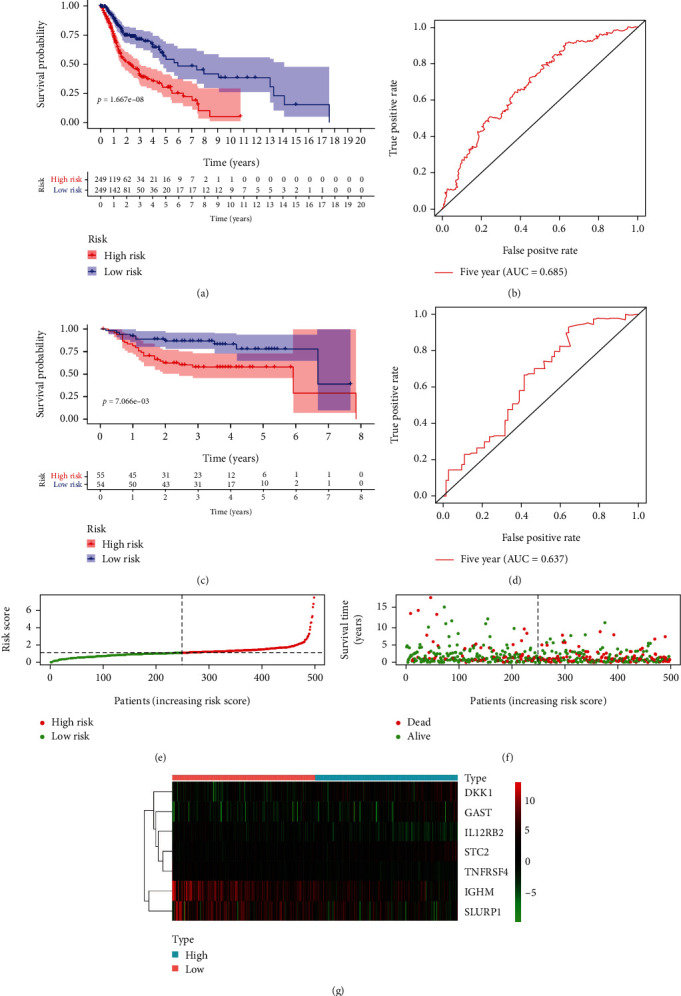
Development and validation of prognostic signature derived from immune-related genes. (a) K-M analysis of the effect of the prognostic signature on OS of HNSCC patients in TCGA database. (b) ROC curves of the prognostic signature in TCGA database. (c) K-M analysis of the effect of the prognostic signature on OS of HNSCC patients in the GEO database. (d) ROC curves of the prognostic signature in the GEO database. (e) The RS distribution in HNSCC patients. (f) The survival status of HNSCC patients. (g) Heatmap of the expression of nine immune-related genes in the low-risk and high-risk groups.

Moreover, individuals with HNSCC were categorized into the low-risk group (*n* = 249) and the high-risk group (*n* = 249) as per the median RS. HNSCC patients at high risk showed a worse survival in the K-M analysis, in comparison to the patients at low risk (*P* < 0.001) (Figure 4(a)), with the AUC of 0.685 for the 5-year ROC curve (Figure 4(b)), indicating a certain predictive value of the signature in predicting the survival of individuals with HNSCC. Meanwhile, this IRG-based signature was validated in the GEO database (GSE27020) of 109 HNSCC patients who were also grouped into the low-risk group (*n* = 54) and the high-risk group (*n* = 55). Consistent with TCGA database, the K-M analysis of the GEO data exhibited that high-risk HNSCC individuals presented a worse survival in comparison with the low-risk group (*P* < 0.05) (Figure 4(c)), with the AUC of 0.637 for 5-year ROC curve (Figure 4(d)).

Additionally, there were more deaths in HNSCC patients with the elevation in the value of RS (Figures 4(e) and 4(f)), and the seven genes showed differences in mRNA expression across the two groups in the heat map (Figure 4(g)).

### 3.3. Use of IRG-Based Signature as an Independent Prognostic Marker for HNSCC

**Figure 5:**
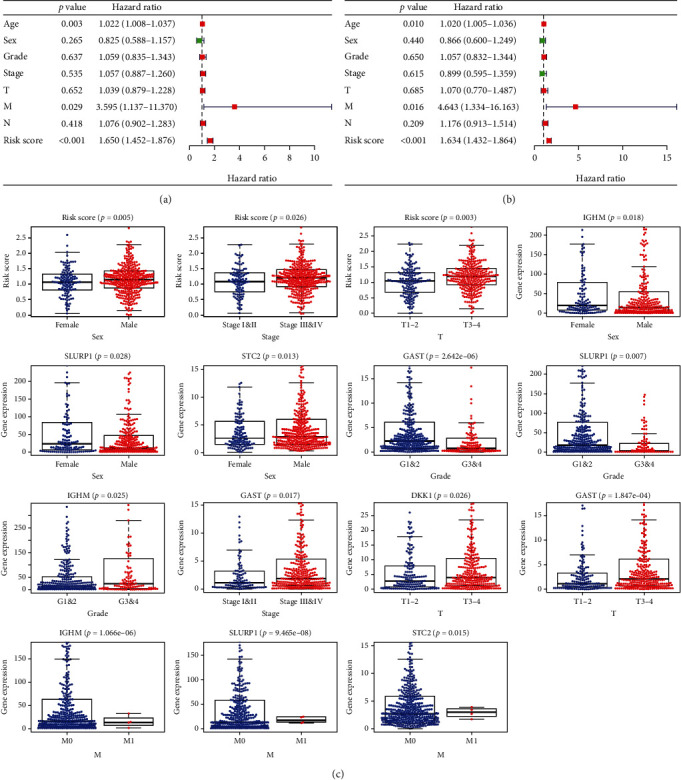
Correlations between the prognostic signature and clinical characteristics of HNSCC. (a) Forest plot of univariate Cox analysis. (b) Forest plot of multivariate Cox analysis. (c) Correlations between the risk score of expression of the seven genes and clinical characteristics.

Univariate and multivariate Cox regression analyses were conducted for assessing the correlations between the IRG-based signature and clinical variables (age, gender, grade, tumor stage, and TNM stage). The findings indicated that OS of HNSCC patients was significantly associated with age (HR = 1.022, 95%CI = 1.008–1.037, *P* = 0.003), M stage (HR = 3.595, 95%CI = 1.137–11.370, *P* = 0.029), and the RS calculated from the IRG-based signature (HR = 1.650, 95%CI = 1.452–1.876, *P* < 0.001) (Figure 5(a)) in univariate Cox regression analysis and also with age (HR = 1.020, 95%CI = 1.005–1.036, *P* = 0.010), M stage (HR = 4.643, 95%CI = 1.334–16.163, *P* = 0.016), and the RS (HR = 1.634, 95%CI = 1.432–1.864, *P* < 0.001) in multivariate Cox regression analysis (Figure 5(b)).

Furthermore, the associations between clinicopathological parameters and the RS and associations between the seven genes and clinical variables were also evaluated (Table 3, Figure 5(c)). The results revealed that the male gender, high tumor stage, and T stage were linked to a greater value of RS. In addition, mRNA expression levels of *IGHM* and *SLURP1* appeared to be elevated in females in comparison to males. The mRNA expression level of STC2 appeared to be elevated in males when compared with females, and a high pathological grade was correlated with lower mRNA expression of *GAST* and *SLURP1*. The results also suggested that higher mRNA expression of *IGHM* was remarkably linked to a high grade. Greater mRNA expression of *GAST* was significantly linked to a more advanced tumor stage. The elevated mRNA expression level of *DKK1* and *GAST* was correlated with the advanced T stage. Moreover, lower mRNA expression of *IGHM*, as well as higher mRNA expression of *SLURP1* and *STC2*, was correlated with the advanced M stage.

### 3.4. Construction of a Predictive Nomogram

**Figure 6:**
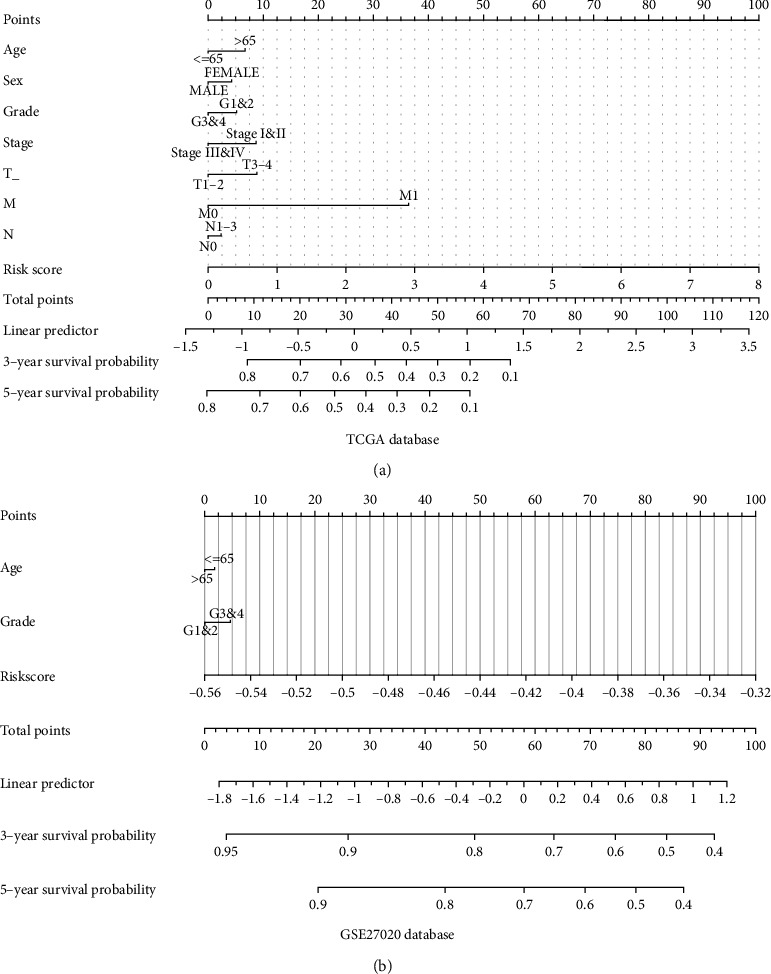
Nomogram for the prediction of survival for patients with HNSCC. (a) Nomogram for the prediction of survival at 3 and 5 years in TCGA database. (b) Nomogram for the prediction of survival at 3 and 5 years in the GSE27020 database.

The clinical variables and RS were included in the nomogram. As indicated in the nomogram, the RS had the largest contribution to survival of patients with HNSCC both in TCGA and GSE27020 databases (Figures 6(a) and 6(b)).

### 3.5. Somatic Mutation Analysis

**Figure 7:**
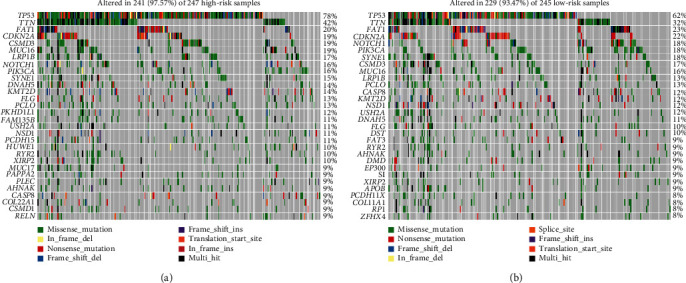
Evaluation of somatic mutation. (a) The mutation profile of the top 30 mutation genes in high-risk patients. (b) The mutation profile of the top 30 mutation genes in low-risk patients.

We obtained somatic mutation profiles of 528 patients in TCGA database. Around 241 (97.57%) and 229 (93.47%) samples possessed somatic mutations in the high-risk and low-risk groups, respectively. The top 30 mutated genes for high-risk and low-risk groups are shown in Figures 7(a) and 7(b). The results indicated that the *TP53* mutated most frequently approximately accounting for 78% and 62% in the high-risk and low-risk groups, respectively.

### 3.6. Immune Microenvironment Analysis

**Figure 8:**
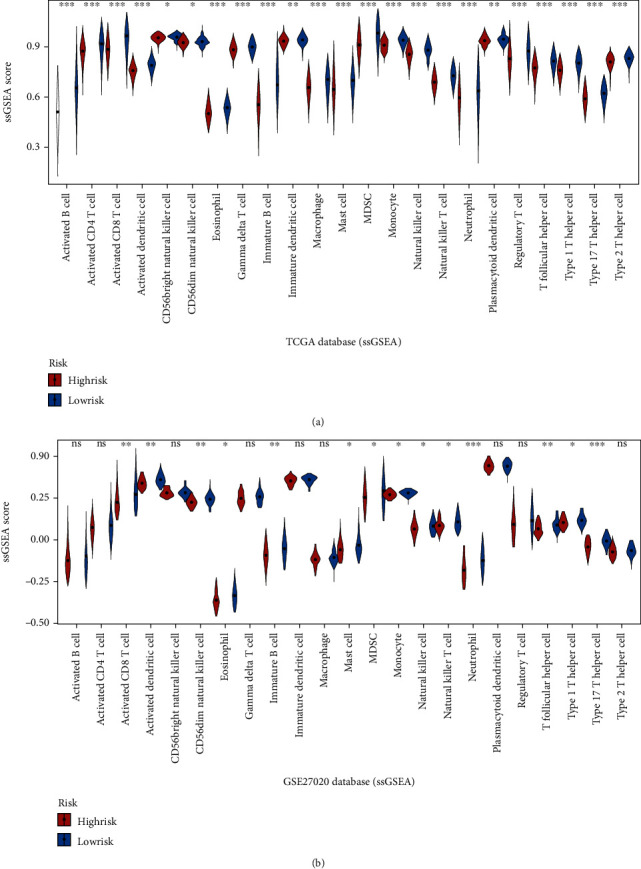
Evaluation of tumor immune microenvironment. Violin plots showed the detailed differences for 23 immune cells between high-risk and low-risk patients in TCGA database (a) and GSE27020 database (b). ^∗^*P* < 0.05,  ^∗∗^*P* < 0.01, and^∗∗∗^*P* < 0.001.

The association between 23 immune cells infiltration differences and different risk groups was analyzed in TCGA and GSE27020 databases. Patients in the low-risk group showed higher infiltration levels of these 23 immune cells in TCGA database (Figure 8(a)), and patients in the low-risk group were more correlated with the infiltration of activated CD8 T cell, activated dendritic cell, CD56dim natural killer cell, eosinophil, immature B cell, mast cell, MDSC, monocyte, natural killer cell, natural killer T cell, neutrophil, T follicular helper cell, type 1 T helper cell, and type 17 T helper cell in the GSE27020 database (Figure 8(b)).

In addition, the relationship between immune pathway scores and RS were analyzed in order to better explore the immune-related biological functions. Functions with a correlation greater than 0.2 and *P* < 0.05 are shown in Supplementary Figure 1. The results indicated that 14 immune-related pathways were correlated negatively with the RS in TCGA database (Supplementary Figure 1A). In the GSE27020 database, 8 immune-related pathways were correlated negatively with the RS, while 1 was correlated positively (Supplementary Figure 1B). These immune-related pathway scores vary with increasing levels of RS, implying that an imbalance in these pathways is closely related to tumor development.

### 3.7. Prediction of Clinical Application

**Figure 9:**
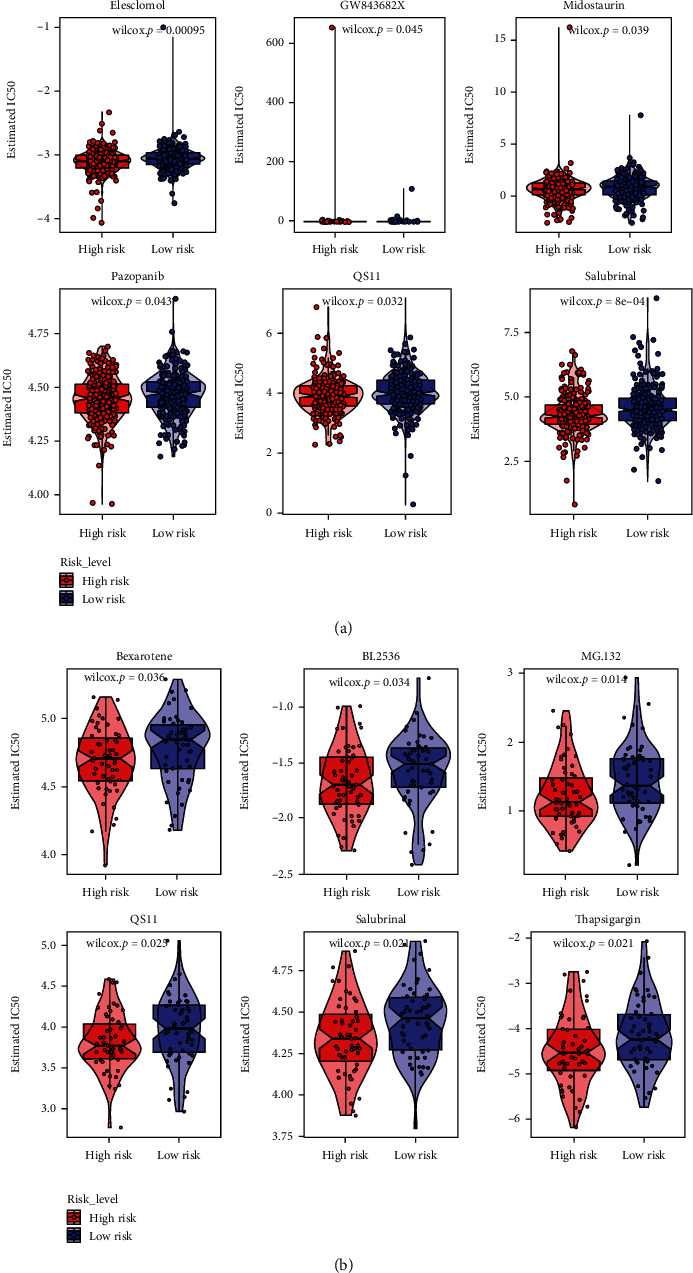
Prediction of clinical application. Correlations between the risk score of the prognostic signature and the efficacy of common chemotherapeutics in TCGA database (a) and GSE27020 database (b).

The association of risk with the therapeutic efficacy of common chemotherapeutic agents in HNSCC was also studied. The findings exhibited that the low-risk HNSCC patients presented increased sensitivity to Elesclomol, GW843682X, Midostaurin, Pazopanib, QS11, and Salubrinal in TCGA database (Figure 9(a)), and the low-risk group was more likely with higher sensitivity of Bexarotene, BI.2536, MG.132, QS11, Salubrinal, and Thapsigargin in the GSE27020 database (Figure 9(b)). The results indicated that HNSCC patients with low risk represented higher sensitivity to chemotherapy.

### 3.8. Investigation of the Expression of the Seven IRGs

**Figure 10:**
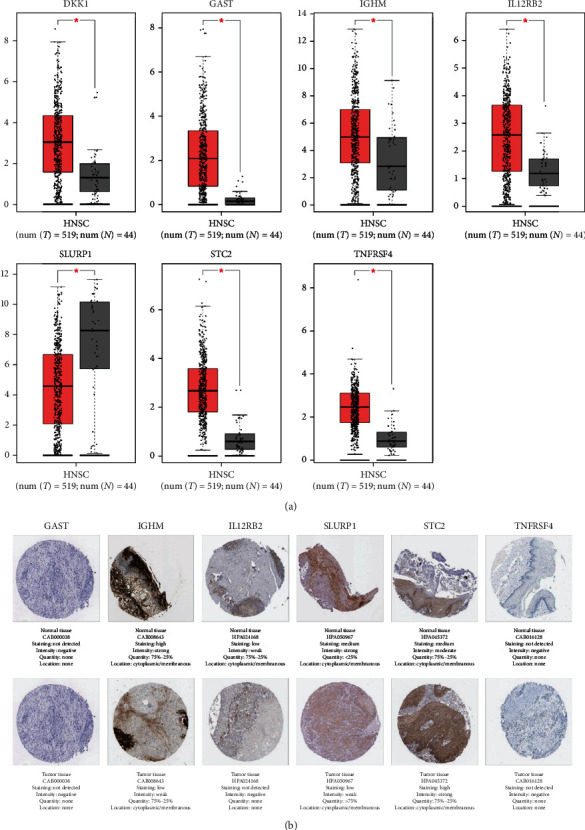
The expression of the seven IRGs. (a) The mRNA expressions of the hub genes from the GEPIA database. (b) Validation of the hub genes on a translational level using the HPA database.

The expression of the seven IRGs in HNSCC was explored with the help of the GEPIA database. The expression levels of the seven IRGs varied remarkably across cancerous and healthy tissues (Figure 10(a)). However, to validate these findings, more experimental analyses were required. Moreover, the HPA database was employed to investigate the expression of the seven IRGs at the translation level. Among the seven IRGs, expressions of *IGHM* and *SLURP1* were lower in the HNSCC tissues. Moreover, *STC2* showed higher expression in HNSCC by immunohistochemistry. No remarkable variations were observed in the expressions of *GAST*, *IL12RB2*, and *TNFRSF4* across normal and HNSCC tissues, while *DKK1* was not detected by immunohistochemistry in the HPA database (Figure 10(b)). However, to further validate the translational relevance of the seven IRGs on HNSCC, more clinical analyses on HNSCC samples are needed.

## 4. Discussion

During this research, an IRG-based signature was established, which was capable of anticipating the clinical landscapes of HNSCC patients and correlated with clinicopathological characteristics of affected individuals, the numbers of tumor-infiltrating immune cells, and the efficacy of common chemotherapeutics. These findings suggested that this signature may be valuable for predicting HNSCC-related prognosis and provide good clinical application in immunotherapy and chemotherapy.

The IRG-based signature consisted of seven genes (i.e., *DKK1*, *GAST*, *IGHM*, *IL12RB2*, *SLURP1*, *STC2*, and *TNFRSF4*). Among them, *DKK1* is a member of the DKK family and regulates cell proliferation, migration, and apoptosis in various tumor tissues through *β*-catenin-dependent and *β*-catenin-independent mechanisms [28]. Moreover, as a tumor suppressor gene, *DKK1* causes apoptosis and suppresses cell proliferation [29]. Gao et al. suggested that elevated *DKK1* expression levels can predict poor prognosis in HNSCC patients [30]. *STC2* regulates tumor cell proliferation, apoptosis, and angiogenesis and is also vital for the invasiveness and metastasis of HNSCC [31]. *IL12RB2* is a subunit of the IL-12 receptor, and an increased ratio of *IL12RB2*-positive tumor-infiltrating lymphocytes is indicative of a good prognosis in laryngeal cancer [32]. *SLURP1* belongs to the Ly6/uPAR family that lacks a GPI-anchoring signal sequence and is associated with a poor prognosis of HNSCC [33]. Furthermore, one of the tumor necrosis factor receptors, *TNFRSF4*, could be a useful target for immunotherapy of HNSCC [34]. Although there are no published reports on *GAST* and *IGHM* for HNSCC, these genes may be related to tumorigenesis and development [35, 36]. In general, these previous findings emphasize the importance of these seven genes in HNSCC prognosis prediction. Furthermore, the expression levels of *GAST*, *IL12RB2*, and *TNFRSF4* in HNSCC samples appeared to be elevated in healthy tissues from the GEPIA database, while no apparent variations were observed between cancerous and healthy tissues from the HPA data. Except for *SLURP1* and *STC2*, *IGHM* expression in HNSCC tissues was remarkably increased compared to that in healthy samples from the GEPIA database, which was inconsistent with the HPA database. This could be due to abnormal methylation. However, further experimentation is required to confirm this finding.

In the multivariate analysis for the associations between clinicopathological factors and the risk IRG-based signature, a high-immune RS was linked to a high tumor stage and T stage. Also, the signature predicted the possible clinical features of HNSCC subjects, likely by regulating the tumor immune microenvironment. The tumor-infiltrating immune cells are known to be correlated with the progression and OS of HNSCC subjects [37], and a high level of infiltrating immune cells is often a good predictor for the OS of patients [38, 39]. Therefore, the risk IRG-based signature is expected to correlate with infiltrating immune cells. As expected, low-risk HNSCC patients had increased infiltration rates of 23 immune cells, indicating the effectiveness of immunotherapy in the low-risk category compared with that in the high-risk category. Owing to the importance of chemotherapy in HNSCC, the IC_50_ values of various chemotherapeutic agents were compared in the two groups. A lower RS was linked to a higher IC_50_ of QS11 and Salubrinal in both TCGA and GSE27020 databases. The QS11 is an inhibitor of ADP-ribosylation factor GTPase-activating protein 1, which modulates Wnt/*β*-catenin signaling through an effect on protein trafficking [40]; and the Salubrinal is a selective cell complex inhibitor that inhibits endoplasmic reticulum stress-mediated apoptosis. Despite these two drugs are not commonly used as chemotherapy drugs for HNSCC, the findings of this study may be valuable for future research.

This study had some limitations. (i) Owing to limited HNSCC samples in TCGA and GSE27020 databases, an issue of the time period was evident in this study. (ii) The analyses were performed using publicly available data from retrospective studies, and the outcomes must be validated in further research with larger samples and functional experiments. (iii) There is a need for further exploration of other possible predictive factors linked to clinical outcomes in HNSCC. (iv) There is a need for further investigation of the mechanisms underlying the functions of the IRG-based signature in HNSCC. Bioinformatics analysis with a specific reference value was used as a basis to conclude this study. Further corresponding molecular experiments are required to validate these findings.

To conclude, a risk signature based on seven IRGs (*DKK1*, *GAST*, *IGHM*, *IL12RB2*, *SLURP1*, *STC2*, and *TNFRSF4*) was developed. This signature serves as a potential biological marker and treatment target for immunotherapy and chemotherapy of HNSCC. These findings may facilitate future studies on the molecular mechanisms of HNSCC.

## Figures and Tables

**Table 1:** Comparison of characteristics between TCGA database and the GEO database (GSE27020).

Characteristic	TCGA		GSE27020		*P* ^∗^
	Frequency	Deaths (%)	Frequency	Deaths (%)	
Total	528	170 (32.20)	109	34 (31.19)	
Age (years)					0.063
≤65	345	99 (28.70)	61	19 (31.15)	
>65	182	71 (39.01)	48	15 (31.25)	
Unknown	1	0			
Sex					—
Male	386	114 (29.53)	—	—	
Female	142	56 (39.44)	—	—	
Grade					0.023
G1-G2	374	120 (32.09)	91	29 (31.87)	
G3-G4	132	43 (32.58)	16	5 (31.25)	
Unknown	22	7 (31.82)	2	0	
Stage					—
Stage I-stage II	120	40 (33.33)	—	—	
Stage III-stage IV	394	130 (32.99)	—	—	
Unknown	14	0			
T					—
T1-T2	189	53 (28.04)	—	—	
T3-T4	323	117 (36.22)	—	—	
Unknown	16	0			
M					—
M0	496	166 (33.47)	—	—	
M1	6	3 (50.00)	—	—	
Unknown	26	1 (3.85)			
N					—
N0	246	88 (35.77)	—	—	
N1-N3	260	81 (31.15)	—	—	
Unknown	22	1 (4.55)			

TCGA: The Cancer Genome Atlas; GEO: Gene Expression Omnibus. ^∗^Chi-square test for the comparison of characteristics between TCGA database and the GSE27020 database for each clinical variable.

**Table 2:** The detailed information of the immune-related gene signature for the survival of HNSCC patients.

Gene name	Coef	HR (95% CI)	*P* value
*DKK1*	0.006062	1.006 (0.998-1.014)	0.118
*GAST*	0.010886	1.011 (1.001-1.021)	0.024
*IGHM*	-0.000928	0.999 (0.998-1.000)	0.108
*IL12RB2*	-0.051088	0.950 (0.894-1.010)	0.099
*SLURP1*	-0.001863	0.998 (0.997-1.000)	0.012
*STC2*	0.025190	1.026 (1.006-1.046)	0.011
*TNFRSF4*	-0.089341	0.915 (0.831-1.007)	0.069

HNSCC: head and neck squamous cell carcinoma; Coef: regression coefficient value; HR: hazard ratio; CI: confidence interval.

**Table 3:** Correlations between the seven immune-related genes and clinical characteristics.

Gene name	Age (*P* value)	Sex (*P* value)	Grade (*P* value)	Stage (*P* value)	T (*P* value)	M (*P* value)	N (*P* value)
*DKK1*	1.314 (0.190)	-0.346 (0.730)	-1.882 (0.062)	-1.725 (0.086)	-2.233 (0.026)	-0.502 (0.649)	-1.181 (0.238)
*GAST*	-1.550 (0.123)	-0.787 (0.432)	4.759 (<0.001)	-2.389 (0.017)	-3.773 (<0.001)	0.013 (0.990)	-0.107 (0.915)
*IGHM*	0.207 (0.836)	-2.370 (0.018)	-2.271 (0.025)	0.009 (0.993)	1.094 (0.275)	5.116 (<0.001)	-1.114 (0.266)
*IL12RB2*	-0.347 (0.729)	1.676 (0.095)	-0.499 (0.618)	1.820 (0.071)	1.411 (0.160)	0.459 (0.677)	1.261 (0.208)
*SLURP1*	0.071 (0.943)	2.221 (0.028)	2.717 (0.007)	1.622 (0.107)	0.632 (0.528)	5.777 (<0.001)	1.517 (0.130)
*STC2*	-1.203 (0.230)	-2.498 (0.013)	-1.658 (0.100)	-1.140 (0.256)	-0.647 (0.518)	3.320 (0.015)	0.085 (0.932)
*TNFRSF4*	1.154 (0.250)	-1.014 (0.311)	-1.484 (0.140)	-0.900 (0.368)	1.407 (0.161)	0.662 (0.535)	-1.381 (0.169)

## Data Availability

The datasets analyzed during the current study are available in TCGA database (https://portal.gdc.cancer.gov/), ImmPort database (https://immport.niaid.nih.gov/), GEO database (https://www.ncbi.nlm.nih.gov/gds/), TIMER (https://cistrome.shinyapps.io/timer/), STRING biological database (https://string-db.org/), GEPIA database (http://gepia.cancer-pku.cn/), and HPA database (https://www.proteinatlas.org/).

## Supplementary Materials

Supplementary Materials Supplementary Figure 1: immune-related biological functions.

## References

[1] Liu H., Li G., Sturgis E. M. (2022). Genetic variants in CYP2B6 and HSD17B12 associated with risk of squamous cell carcinoma of the head and neck. *International Journal of Cancer*.

[2] Siegel R. L., Miller K. D., Fuchs H. E., Jemal A. (2022). Cancer statistics, 2022. *CA: a Cancer Journal for Clinicians*.

[3] Leemans C. R., Snijders P. J. F., Brakenhoff R. H. (2018). The molecular landscape of head and neck cancer. *Nature Reviews. Cancer*.

[4] Johnson D. E., Burtness B., Leemans C. R., Lui V. W. Y., Bauman J. E., Grandis J. R. (2020). Head and neck squamous cell carcinoma. *Nature reviews Disease primers*.

[5] Badr M., Jöhrens K., Allgäuer M. (2019). Morphomolecular analysis of the immune tumor microenvironment in human head and neck cancer. *Cancer Immunology, Immunotherapy*.

[6] Chen S. M. Y., Krinsky A. L., Woolaver R. A., Wang X., Chen Z., Wang J. H. (2020). Tumor immune microenvironment in head and neck cancers. *Molecular Carcinogenesis*.

[7] Fu C., Zhao H., Wang Y. (2016). Tumor-associated antigens: Tn antigen, sTn antigen, and T antigen. *Hla*.

[8] Ferris R. L., Hunt J. L., Ferrone S. (2005). Human leukocyte antigen (HLA) class I defects in head and neck cancer: molecular mechanisms and clinical significance. *Immunologic Research*.

[9] Canning M., Guo G., Yu M. (2019). Heterogeneity of the head and neck squamous cell carcinoma immune landscape and its impact on immunotherapy. *Frontiers in Cell and Development Biology*.

[10] Fiedler M., Weber F., Hautmann M. G., Bohr C., Reichert T. E., Ettl T. (2020). Infiltrating immune cells are associated with radiosensitivity and favorable survival in head and neck cancer treated with definitive radiotherapy. *Oral Surgery, Oral Medicine, Oral Pathology, Oral Radiology*.

[11] Duhen R., Ballesteros-Merino C., Frye A. K. (2021). Neoadjuvant anti-OX40 (MEDI6469) therapy in patients with head and neck squamous cell carcinoma activates and expands antigen-specific tumor- infiltrating T cells. *Nature Communications*.

[12] Peng Y., Xiao L., Rong H. (2021). Single-cell profiling of tumor-infiltrating TCF1/TCF7^+^ T cells reveals a T lymphocyte subset associated with tertiary lymphoid structures/organs and a superior prognosis in oral cancer. *Oral Oncology*.

[13] Kaidar-Person O., Gil Z., Billan S. (2018). Precision medicine in head and neck cancer. *Drug Resistance Updates*.

[14] Horton J. D., Knochelmann H. M., Day T. A., Paulos C. M., Neskey D. M. (2019). Immune evasion by head and neck cancer: foundations for combination therapy. *Trends Cancer*.

[15] Cohen E. E. W., Bell R. B., Bifulco C. B. (2019). The Society for Immunotherapy of Cancer consensus statement on immunotherapy for the treatment of squamous cell carcinoma of the head and neck (HNSCC). *Journal for Immunotherapy of Cancer*.

[16] Yao Y., Yan Z., Lian S. (2020). Prognostic value of novel immune-related genomic biomarkers identified in head and neck squamous cell carcinoma. *Journal for Immunotherapy of Cancer*.

[17] Chen Y., Li Z. Y., Zhou G. Q., Sun Y. (2021). An immune-related gene prognostic index for head and neck squamous cell carcinoma. *Clinical Cancer Research*.

[18] Zhang Y., Chen P., Zhou Q. (2021). A novel immune-related prognostic signature in head and neck squamous cell carcinoma. *Frontiers in Genetics*.

[19] Liu T., Wu H., Qi J., Qin C., Zhu Q. (2020). Seven immune-related genes prognostic power and correlation with tumor- infiltrating immune cells in hepatocellular carcinoma. *Cancer Medicine*.

[20] Wang Z., Song Q., Yang Z., Chen J., Shang J., Ju W. (2019). Construction of immune-related risk signature for renal papillary cell carcinoma. *Cancer Medicine*.

[21] Ritchie M. E., Phipson B., Wu D. (2015). Limma powers differential expression analyses for RNA-sequencing and microarray studies. *Nucleic Acids Research*.

[22] Li C. Y., Cai J.-H., Tsai J. J. P., Wang C. C. N. (2020). Identification of hub genes associated with development of head and neck squamous cell carcinoma by integrated bioinformatics analysis. *Frontiers in Oncology*.

[23] Yang J., Xie K., Li C. (2020). Immune-related genes have prognostic significance in head and neck squamous cell carcinoma. *Life Sciences*.

[24] Hu Z., Zhou J., Li Y. (2022). Peripheral immune signature resembles tumor microenvironment and predicts clinical outcomes in head and neck squamous cell carcinoma. *Frontiers in Immunology*.

[25] Liang J. X., Chen Q., Gao W. (2022). A novel glycosylation-related gene signature predicts survival in patients with lung adenocarcinoma. *BMC Bioinformatics*.

[26] Doncheva N. T., Morris J. H., Gorodkin J., Jensen L. J. (2019). Cytoscape StringApp: network analysis and visualization of proteomics data. *Journal of Proteome Research*.

[27] Yu G., Wang L. G., Han Y., He Q. Y. (2012). clusterProfiler: an R package for comparing biological themes among gene clusters. *OMICS*.

[28] Zhu G., Song J., Chen W. (2021). Expression and role of Dickkopf-1 (Dkk1) in tumors: from the cells to the patients. *Cancer Management and Research*.

[29] Niu J., Li X. M., Wang X. (2019). DKK1 inhibits breast cancer cell migration and invasion through suppression of *β*-catenin/MMP7 signaling pathway. *Cancer Cell International*.

[30] Gao H., Li L., Xiao M. (2018). Elevated DKK1 expression is an independent unfavorable prognostic indicator of survival in head and neck squamous cell carcinoma. *Cancer Management and Research*.

[31] Ma H. F., Lv G. X., Zhang D. H. (2020). miR-381 mediates the development of head and neck squamous cell carcinoma via targeting STC2. *Oncotargets and Therapy*.

[32] Tao Y., Gross N., Liu Y. (2017). A high ratio of IL-12R*β*2-positive tumor-infiltrating lymphocytes indicates favorable prognosis in laryngeal cancer. *Oral Oncology*.

[33] Zheng H., Liu H., Lu Y., Li H. (2021). Identification of a novel signature predicting overall survival in head and neck squamous cell carcinoma. *Frontiers in Surgery*.

[34] Loick S. M., Fröhlich A., Gabrielpillai J. (2022). DNA methylation and mRNA expression of OX40 (TNFRSF4) and GITR (TNFRSF18, AITR) in head and neck squamous cell carcinoma correlates with HPV status, mutational load, an interferon-*γ* signature, signatures of immune infiltrates, and survival. *Journal of Immunotherapy*.

[35] Ness-Jensen E., Bringeland E. A., Mattsson F. (2020). Hypergastrinemia is associated with an increased risk of gastric adenocarcinoma with proximal location: a prospective population-based nested case-control study. *International Journal of Cancer*.

[36] Lu Z., Gao Y. (2021). Screening differentially expressed genes between endometriosis and ovarian cancer to find new biomarkers for endometriosis. *Annals of Medicine*.

[37] Almangush A., Leivo I., Mäkitie A. A. (2020). Overall assessment of tumor-infiltrating lymphocytes in head and neck squamous cell carcinoma: time to take notice. *Acta Oto-Laryngologica*.

[38] Spector M. E., Bellile E., Amlani L. (2019). Prognostic value of tumor-infiltrating lymphocytes in head and neck squamous cell carcinoma. *JAMA Otolaryngology. Head & Neck Surgery*.

[39] Xu Q., Wang C., Yuan X., Feng Z., Han Z. (2017). Prognostic value of tumor-infiltrating lymphocytes for patients with head and neck squamous cell carcinoma. *Translational Oncology*.

[40] Singh M. K., Gao H., Sun W. (2015). Structure-activity relationship studies of QS11, a small molecule Wnt synergistic agonist. *Bioorganic & Medicinal Chemistry Letters*.

