# Correction to “Seven Immune‐Related Genes’ Prognostic Value and Correlation with Treatment Outcome in Head and Neck Squamous Cell Carcinoma”

**DOI:** 10.1155/mi/9801859

**Published:** 2025-12-17

R. Mu, Y. Shen, C. Guo, X. Zhang, H. Yang, and H. Yang, “Seven Immune‐Related Genes’ Prognostic Value and Correlation with Treatment Outcome in Head and Neck Squamous Cell Carcinoma,” *Mediators of Inflammation*, 2023, https://doi.org/10.1155/2023/8533476.

In the article, there is an error in Figure 10b. The normal and tumor GAST images were inadvertently duplicated during the preparation of the figure and the correct Figure 10 is shown below:

Figure 10The expression of the seven IRGs. (a) The mRNA expressions of the hub genes from the GEPIA database. (b) Validation of the hub genes on a translational level using the HPA database.

**Figure:**
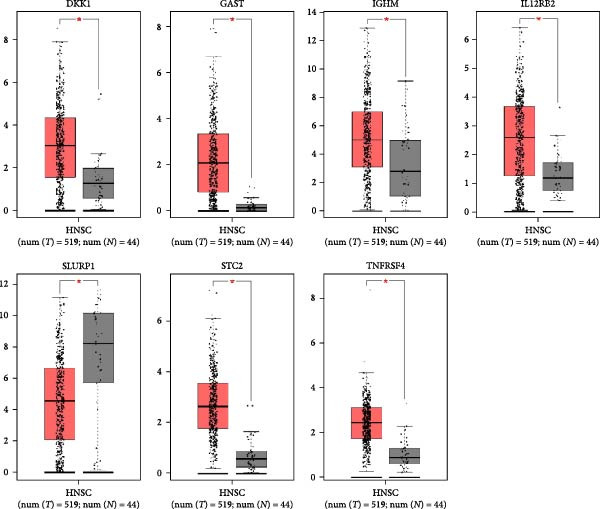
(a)

**Figure:**
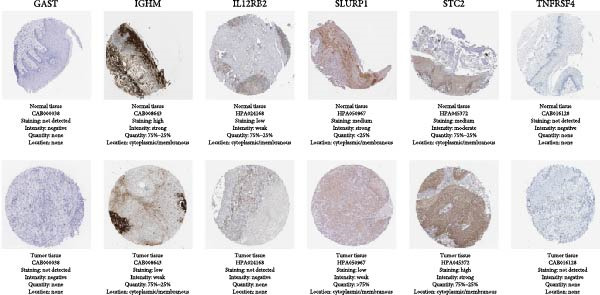
(b)

We apologize for this error.

