# Supplementary material for: Seven Immune-Related Genes' Prognostic Value and Correlation with Treatment Outcome in Head and Neck Squamous Cell Carcinoma
Source: Mediators Inflamm. 2023 Apr 20;2023:8533476. doi: 10.1155/2023/8533476 (PMC11401713; doi:10.1155/2023/8533476)
Supplement: Supplementary Materials — Supplementary Figure 1: immune-related biological functions. [file 8533476.f1.docx]

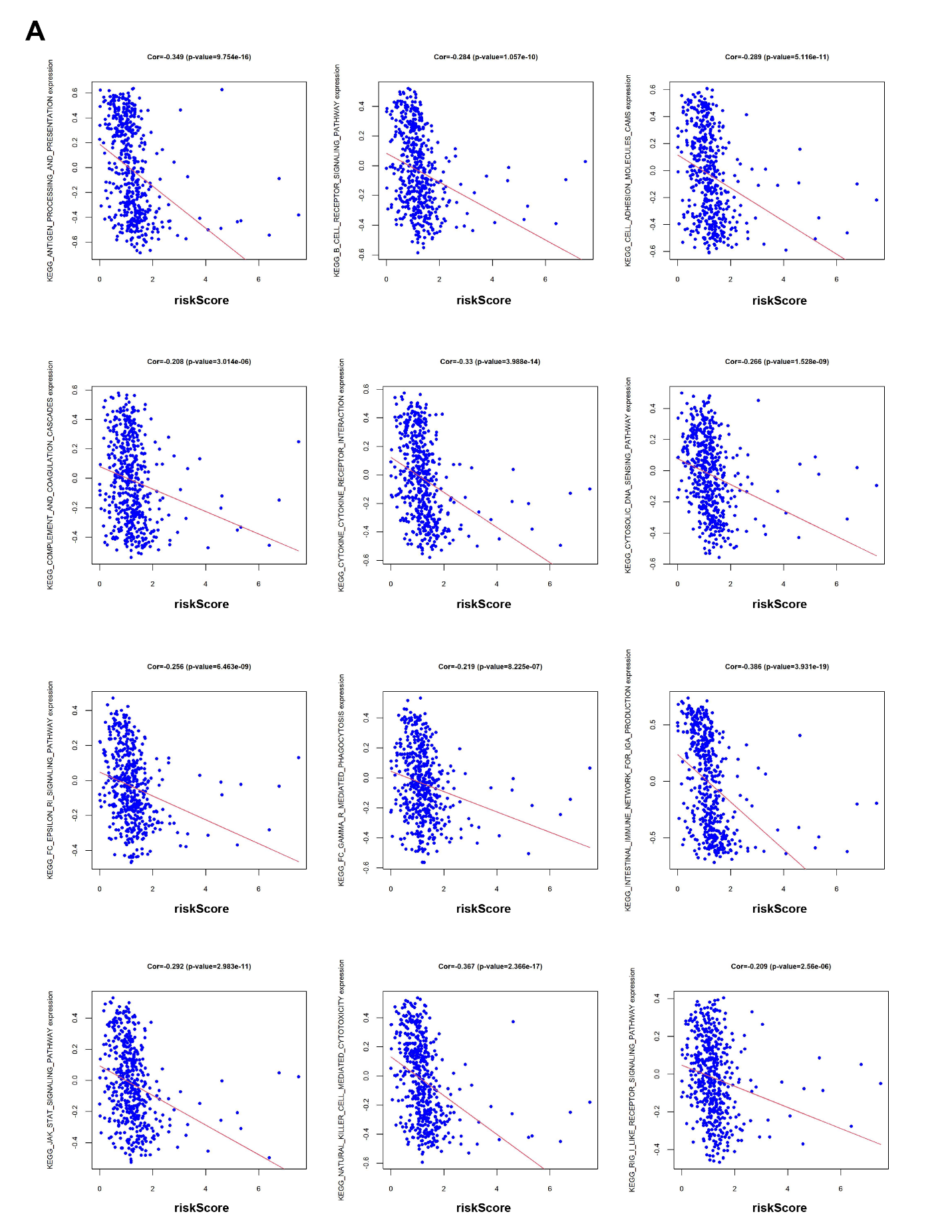


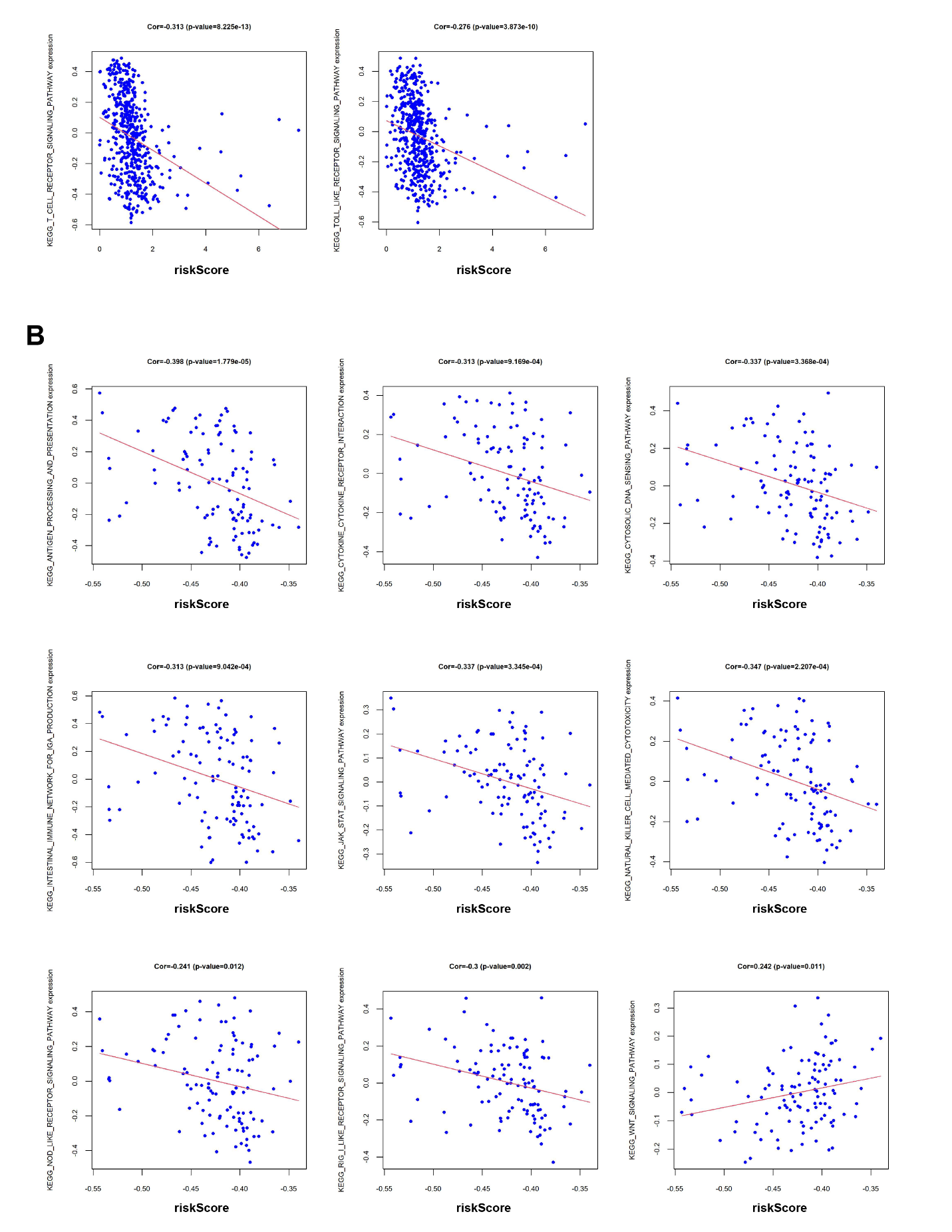


**Supplementary Figure 1.** Immune-related biological functions. (A) Relation between the immune pathway scores and risk scores with a correlation greater than 0.2 and *p* < 0.05 in the TCGA database. (B) Relation between the immune pathway scores and risk scores with a correlation greater than 0.2 and *p* < 0.05 in the GSE27020 database.
